# Sexual health literacy and its related factors among couples: A population-based study in Iran

**DOI:** 10.1371/journal.pone.0293279

**Published:** 2023-11-01

**Authors:** Hadis Shahrahmani, Nourossadat Kariman, Zohreh Keshavarz, Atefeh Ahmadi, Malihe Nasiri

**Affiliations:** 1 Department of Midwifery and Reproductive Health, Student Research Committee, School of Nursing and Midwifery, Shahid Beheshti University of Medical Sciences, Tehran, Iran; 2 Department of Midwifery and Reproductive Health, Midwifery and Reproductive Health Research Center, School of Nursing and Midwifery, Shahid Beheshti University of Medical Sciences, Tehran, Iran; 3 Department of Counselling in Midwifery, Nursing Research Center, School of Nursing and Midwifery, Kerman University of Medical Sciences, Kerman, Iran; 4 Department of Biostatistics, Midwifery and Reproductive Health Research Center, School of Nursing and Midwifery, Shahid Beheshti University of Medical Sciences, Tehran, Iran; Hamadan University of Medical Sciences School of Dentistry, ISLAMIC REPUBLIC OF IRAN

## Abstract

Sexual health literacy is one of the factors that affect sexual health. Several factors can influence sexual health literacy. As a result, the current study was carried out to determine sexual health literacy and its associated factors among Iranian couples. In 2022, 410 couples of reproductive age were referred to comprehensive health service centers and private clinics in Kerman city for the descriptive-analytical study. The study questionnaires included sexual health literacy for adults, sexual knowledge and attitude scale, marital intimacy scale, sexual intimacy scale, depression anxiety stress scale, sexual self-efficacy, the multidimensional scale of perceived social support, and socioeconomic status. Finally, multiple linear stepwise regression models were used to determine the factors related to sexual health literacy using the SPSS software version 22. According to the findings of this study, the mean(SD) of sexual health literacy in couples was 68.76(12.96), which was a desirable level. Furthermore, the findings revealed that sexual self-efficacy (B = 0.649، P<0.001), sexual knowledge and attitude (B = 0.217، P<0.001), the ability to identify reliable and non-reliable sources (B = -3.116، P<0.001), feelings of shame and embarrassment for obtaining sexual information (B = 1.860، P = 0.011), social support (B = 0.127، P<0.001) and the obscenity of sexual issues in family (B = 1.764، P = 0.015) were the final predictors of sexual health literacy in couples. It is suggested that researchers and health managers consider these factors when designing interventions to promote sexual health literacy.

## Introduction

Sexual health, according to the World Health Organization, is essential for achieving physical and emotional health, the well-being of individuals, couples, and families, and the social and economic development of societies and countries [1]. In this regard, sexual health literacy is among the factors related to sexual health [2, 3].

Sexual health literacy is defined as an individual’s knowledge, beliefs, attitudes, motivations, and skills in accessing, comprehending, evaluating, and applying sexual health related information in order to negotiate, judge, and make sexual health-related decisions [4].

A study by Vongxay in Laos in 2019 showed that 65.5% of young people lacked sexual health literacy. According to the study, demographic factors, knowledge about sexual health, and participation in relevant educational classes predict sexual health literacy [5]. The results of a study in Iran showed that 85% of the evaluated couples lacked adequate sexual and reproductive health literacy [6]. Based on the results of this study, majority of couples lacked sufficient skills in comprehending information, obtaining reliable information, analyzing information, and correct applying sexual and reproductive health related information [7].

Previous research has linked low sexual health literacy to poor sexual performance [3], delayed or difficult treatment seeking, low sexual entitlement [7], poor quality of sexual life [2], decreased knowledge, attitude, and self-efficacy towards condom use [8], and an increase in high-risk sexual behaviors [9]. The significance of sexual health literacy is linked to the negative health outcomes caused by lack of knowledge and skills required to engage in preventive health behaviors [10].

Adequate sexual health literacy can improve the ability to understand and evaluate sexual health related risks, postpone the first sexual experience, reduce the number and choose low-risk sexual partners, practice safe sex, reduce unwanted pregnancies and sexually transmitted diseases, promote correct understanding of the duties and responsibilities in sexual relations, improve sexual interactions between couples, improve individual sexual health, and finally improve family and social health [11].

To the best of our knowledge, no study has yet determined the predictive factors of sexual health literacy among couples. Hence, determining the status and predictors of sexual health literacy may be required in order to design interventions to improve sexual health literacy. Therefore, the current study was designed to determine sexual health literacy and its predictors among Iranian couples.

## Methods

### Study population, setting, and sampling method

The present study was a cross-sectional study that was carried out in Kerman, Iran, in 2022. The sampling period lasted from January 2022 to May of 2022. The study population consisted of married couples who met the study’s inclusion criteria. The inclusion criteria were being resident of Kerman city, Iranian nationality, being monogamous, having adequate reading and writing skills, women between the ages of 18 and 49, and men between the ages of 18 and 65. Couples were excluded from the study due to incomplete questionnaires.

The sampling strategy used was multi-stage random sampling. In the following steps, sampling regions were identified. First, comprehensive health service centers and private clinics in Kerman were listed. Then, the centers and clinics were divided into four strata: north, south, east, and west, with two centers and four clinics (Obstetrics and Gynecology and Pediatrics) selected at random from each stratum. Then, based on the covered population, the population in each comprehensive health service center was determined. Samples were selected using the quota sampling method. Due to the uncertainty of the population referring to private clinics, an equal population was considered for all private clinics. As a result, the study included 107 couples from comprehensive health service centers and 106 couples from private clinics. Finally, convenient sampling was used to conduct sequential sampling in comprehensive health service centers and clinics consecutively.

### Sample size determination

Based on the findings of the study by Jamali et al. (2020), the minimum sample size of 385 participants was calculated using the following equation with a standard deviation (σ) for sexual health literacy score of 12 and accuracy (d) of 1.2 and considering α = .05 and z = 1.96. The sample size was increased to 426 (213 women and 213 men), given a 10% drop-out rate [12].


n=zα22σ2d2

### Data collection tools

#### Sexual health literacy for adults (SHELA)

*Outcome measurement*. This questionnaire was designed by Maasoumi et al. (2019) [11]. In the present study, the validity of the questionnaire was examined using face and content validity as well as construct validity (exploratory factor analysis [EFA] and confirmatory factor analysis [CFA]). Face validity of the questionnaire was quantitatively assessed by asking 15 couples to rate questionnaire items. The impact score of all items was above 1.5. Content validity was qualitatively confirmed by ten experts in the fields of midwifery, reproductive health, and sexual health, who also had sufficient experience in the field of tool development. Quantitative content validity was evaluated using the content validity ratio (CVR) and content validity index (CVI). The content validity ratio for all items was excellent (above 0.8), and the item level content validity index (I-CVI) was suitable (above 0.9). This questionnaire’s S-CVI/Ave (scale-level content validity index based on the average method) was 0.98.

Construct validity was evaluated using exploratory factor analysis and confirmatory factor analysis. The exploratory factor analysis indicated that six factors, namely access, reading, comprehension, evaluation, seeking treatment and solving sexual problems, and sexual self-care, explained 41.64% of the variance. The extracted factorial structure was evaluated using confirmatory factor analysis, and fit indicators confirmed the model. The internal consistency of the tool was measured using Cronbach’s alpha, which was between 0.855 and 0.953. The instrument’s Intraclass Correlation Coefficient (ICC) ranged between 0.886 and 0. 967. The sexual health literacy questionnaire has 32 items and six domains, including access skills, reading, comprehension, evaluation, seeking treatment and solving sexual problems, and sexual self-care. The raw score of each person in each domain is obtained from the algebraic sum of scores and is converted into a range of 0 to 100 using the following formula.

$$\frac{Responser^{\prime }s\;raw\;score\;in\;the\;domain-Minimum\;possible\;score\;in\;the\;domain}{Maximum\;possible\;score\;in\;the\;domain-minimum\;possible\;score\;in\;the\;domain}$$

The total sexual health literacy score is obtained by summing up the scores of each domain (based on the range from zero to 100) divided by the total number of domains.

#### Demographic, medical, reproductive, and sexual records questionnaire

A questionnaire for measuring demographic, medical, reproductive, and sexual information includes questions about age, level of education, father’s education level, mother’s education level, job, income, race, marriage pattern, duration of the marriage, number of marriages, number of children, use of drugs, tobacco, and alcohol, number of intercourses per month, contraception method, disease history, drug use history, breastfeeding and pregnancy status, number of pregnancies, ability to identify reliable and non-reliable sources, having a sense of shame and embarrassment for obtaining sexual information, history of participating in sex education classes, obscenity of sexual issues in the family, having the motivation to obtaining sexual information, believing that sexual problems are incurable, fear of intercourse, satisfaction from genitalia shape and appearance, and satisfaction from sexual attractiveness.

Qualitative content validity and face validity methods were used to determine the validity of the questionnaire. The qualitative validity of the questionnaire was confirmed by ten experts in midwifery, reproductive health, and sexual health. In addition, the face validity of the questionnaire was evaluated by asking 15 couples to rate the items in terms of ambiguity and difficulty. This questionnaire does not require reliability.

#### Sexual knowledge and attitude scale

This questionnaire was designed and psychometrically evaluated by Besharat (2005). The questionnaire has 30 items and two dimensions: knowledge and sexual attitude. The score of each domain can range between 15 and 75, and the total score of the questionnaire is between 30 and 150 [13]. Based on the study by Banaei et al. (2021), the Cronbach’s alpha coefficient of the questionnaire was 0.881 [14]. The reliability of this questionnaire was determined in the current study using internal consistency and Cronbach’s alpha coefficient (0.893 for the whole questionnaire, and Cronbach’s alpha was higher than 0.7 for all domains).

#### Marital intimacy scale

"Marital Intimacy Scale" was developed by Walker and Thompson in 1983 [15]. This questionnaire is a 17-item questionnaire that was translated by Sanaei et al. (2000). The minimum possible score is 17, and the maximum is 119. Low, medium, and high intimacy are defined as score ranges of 17–34, 35–84, and 85–119, respectively [16]. The validity and reliability of the questionnaire were confirmed in several studies in Iran [16–18]. The reliability of this questionnaire in the current study was determined using internal consistency and Cronbach’s alpha coefficient (0.97).

#### Sexual intimacy scale

This questionnaire was designed and psychometrically evaluated by Botlani et al. in 2010 [19] based on Bagarozzi’s sexual intimacy questionnaire [20]. The minimum score is 30, and the maximum score is 120. A higher score indicates greater sexual intimacy. This questionnaire does not have subscales. Botlani et al. (2010) reported a Cronbach’s alpha coefficient of 0.81 for the questionnaire [19]. The reliability of this questionnaire in the current study was determined using internal consistency and Cronbach’s alpha coefficient (0.925).

#### Depression anxiety stress scale

Lovibond created this 42-item questionnaire in 1995 [21]. The short form of the questionnaire has 21 questions that measure depression (7 questions), anxiety (7 questions), and stress (7 questions). The minimum and maximum possible scores in each domain are 0 and 21. The reliability of the Persian version of this questionnaire for depression, anxiety, and stress was confirmed using Cronbach’s alpha coefficient as 0.77, 0.79, and 0.78, respectively [22]. The reliability of this questionnaire was determined in the current study using internal consistency and Cronbach’s alpha coefficient (0.946 for the whole questionnaire, and Cronbach’s alpha was higher than 0.7 for all domains).

#### Sexual self-efficacy

This questionnaire was designed by Vaziri & Lotfi Kashani in 2013 based on Schwarzer’s general questionnaire self-efficacy. The questionnaire has ten questions. The range of scores is between 0–30, and a higher score indicates more self-efficacy [23]. The reliability of this questionnaire has been confirmed (Cronbach’s alpha coefficient of 0.90) [24]. The reliability of this questionnaire in the current study was determined using internal consistency and Cronbach’s alpha coefficient (0.906).

#### Multidimensional scale of perceived social support

Zimet designed this questionnaire in 1988 [25]. This questionnaire was psychometrically approved in the study by Salimi et al. (2009). This questionnaire has 12 items. The minimum and maximum scores of the questionnaire are 12 and 84, respectively [26]. The internal reliability of the questionnaire was confirmed in a study using Cronbach’s alpha coefficient of 0.9 for the whole tool [27]. The reliability of this questionnaire in the current study was determined using internal consistency and Cronbach’s alpha coefficient (0.905).

#### Socioeconomic status

This questionnaire was designed by Ghodratnama et al. (2013) and has four domains: education, income, economic class, and housing status, and five main questions. The minimum and maximum scores of the questionnaire are five and 25, respectively. A higher score indicates a better status [28]. The internal reliability of the questionnaire was confirmed in the study of Eslami et al. (2014) (Cronbach’s alpha coefficient of 0.83) [29]. The reliability of this questionnaire in the current study was determined using internal consistency and Cronbach’s alpha coefficient (0.9).

### Data collection techniques

Willing and eligible participants completed data collection forms. The questionnaires were completed in several sessions (face-to-face or online interviews). Each participant completed the questionnaire in two or three stages. To complete the questionnaires online, first, the paper format of the questions was provided to the participants, and the possible questions of the participants were explained to them. Then, participants were instructed on how to complete the questionnaires online. In addition, the couples were assured that their information would remain confidential. The paper-based questionnaires were conducted in person in a separate room during an individual interview with the guidance of male and female researchers. After completing the questionnaires, phone numbers were obtained from the couples. Participants’ information and data were anonymized by assigning unique codes to each participant. Participants’ information was kept confidential. Gifts were given to all participants. Participation in the study was completely voluntary and free of charge.

### Data analysis

This research used the statistical package for social sciences (SPSS) software version 22 (SPSS Inc., Chicago, Illinois) to analyze the data. A significance level of 0.05 was considered in this study. Descriptive statistics included frequency, percentage, mean and standard deviation. Continuous variables are presented as mean and standard deviations (SD), and categorical variables are presented as frequency and percentage. Tables and texts were used to illustrate the findings. Inferential statistics were used to determine the predictors of sexual health literacy, and multiple linear stepwise regression models were used to determine factors related to sexual health literacy. First, univariate linear regression was performed considering sexual health literacy as outcome variable, and the variables mentioned in Table 3 were considered independent variables. To control the effect of potential confounders, independent variables with p value less than 0.2 were entered into the initial multilinear model. In regression analysis, quantitative variables are summarized as coefficients and standard errors. The variance inflation factor (VIF) was used to evaluate multicollinearity. Kolmogorov-Smirnov test was used to determine the normal distribution of quantitative variables. In the case of normal distribution, parametric statistical tests were used; otherwise, non-parametric tests were used.

### Ethical considerations

This study was approved by the ethics committee of Shahid Beheshti University of Medical Sciences (IR.SBMU.RETECH.REC.1400.596). After an oral explanation of the research objectives and other necessary explanations, written informed consent was obtained from the participants. The research assistant signed the consent form after taking the participants’ fingerprints.

## Results

Finally, 205 couples (410 participants) were included in the study. Eight couples were excluded from the study due to incomplete questionnaires. There was no missing data for both the dependent variable and independent variables. The mean(SD) age of the female and male participants was 32.56(7.995), (Range: 18–49) and 36.32(8.034) (Range: 22–58) years, respectively. The mean(SD) duration of marriage(month) was 119.72(102.63), (Range: 1–456) and the mean(SD) number of intercourses per month was 6.59(4.29), (Range: 1–25). Table 1 shows the demographic characteristics and sexual and fertility information of the couples.

**Table 1:** Socio-demographic and sexual and reproductive information of the couples sample, Kerman Iran, 2022.

Domain	Variables	N(%)
**Socio-demographic variables**	**Education**	
	Academic	286 (69/8)
	Non-academic	124 (30/2)
	**Father’s education**	
	Academic	70 (17/07)
	Non-academic	340 (82/93)
	**Mother’s education**	
	Academic	45 (10/98)
	Non-academic	365 (89/02)
	**Job**	
	Employment	270 (65/85)
	Non-employment	140 (34/15)
	I**ncome**	
	Insufficient	111 (27/1)
	Moderate	198 (48‏/3)
	sufficient	101 (24/6)
	**Race**	
	Fars	397 (96/83)
	Non-fars	13 (3/17)
	**Marriage pattern (n = 205 couples)**	
	Traditional	98 (47/80)
	Modern	107 (52/20)
	**Number of marriages**	
	1	398 (97/1)
	2	10 (2/4)
	≥3	2 (0/5)
	**Number of children (n = 205 couples)**	
	0	54 (26/3)
	1	66 (32/2)
	2	57 (27/8)
	3	20 (9/8)
	≥4	8 (3/9)
	**Use of drugs, tobacco, alcohol**	
	Yes	100 (24/4)
	No	310 (75/6)
**Medical, Sexual, and reproductive information variables**	**Contraception method (n = 205 couples)**	
	None	22 (10/7)
	Hormonal **&** None hormonal	78 (38/0)
	Withdrawal	105 (51/2)
	**Disease history**	
	Yes	141 (34/4)
	No	269 (65/6)
	**Drug use history**	
	Yes	78 (19/02)
	No	332 (80/98)
	**Breastfeeding and pregnancy status (n = 205 women)**	
	Pregnant	19 (9/3)
	Breastfeeding	18 (8/8)
	Neither	168 (82)
	**Number of pregnancies (n = 205 women)**	
	0	46 (22/4)
	1	66 (32/2)
	2	48 (23/4)
	≥3	45 (22/0)

The results showed that the mean(SD) score of sexual health literacy was 68.76 (12.960) of 100 and was at a desirable level. Table 2 shows sexual health literacy status and other studied variables.

**Table 2:** Descriptive statistics of sexual health literacy and its related factors in couples sample, Kerman Iran, 2022.

Variables	N(%)	
**Ability to identify reliable and non-reliable sources**		
Yes	117 (28/5)	
Somewhat	229 (55/9)	
No	64 (15/6)	
**Having a sense of shame and embarrassment for obtaining sexual information**		
Yes	72 (17/6)	
Somewhat	138 (33/7)	
No	200 (48/8)	
**History of participating in sex education classes**		
Yes	277 (67/6)	
No	133 (32/4)	
**Obscenity of sexual issues in the family**		
Yes	78 (19/0)	
Somewhat	163 (39/8)	
No	169 (41/2)	
**Having the motivation to Obtain sexual information**		
Yes	186 (45/4)	
Somewhat	144 (35/1)	
No	80 (19/5)	
**Believing that sexual problems are incurable**		
Yes	11 (2/7)	
Somewhat	44 (10/7)	
No	355 (86/6)	
**Fear of intercourse**		
Yes	4 (1/0)	
Somewhat	21 (5/1)	
No	385 (93/9)	
**Scales**	**M(SD)**	**Range**
**Satisfaction from genitalia shape and appearance**	7/74(2/07)	1–10
**Satisfaction from sexual attractiveness**	7/61(2/22)	1–10
**Sexual intimacy**	99/11(13/06)	48–116
**Marital intimacy**	99/19(21/02)	17–119
**Sexual self-efficacy**	18/79(5/45)	0–30
**Sexual Knowledge and Attitude**	123/54(14/06)	81–150
**Depression, Stress, Anxiety**	15/97(12/31)	0–63
**Social support**	61/79(14/20)	12–84
**Socioeconomic status**	11/45(2/70)	5–20
**Sexual health literacy**	68/76(12/96)	7–100

Based on the results of univariate linear regression, age, education, participants’ parental education, socioeconomic status, disease history, ability to identify reliable and non-reliable sources, having a sense of shame and embarrassment for obtaining sexual information, history of participating in sex education classes, obscenity of sexual issues in the family, having the motivation to Obtaining sexual information, satisfaction from genitalia shape and appearance, satisfaction from sexual attractiveness, believing that sexual problems are incurable, sexual knowledge and attitude scale, marital intimacy scale, sexual intimacy scale, depression anxiety stress scale, sexual self-efficacy, and multidimensional scale of perceived social support were significantly related to sexual health literacy (Table 3).

**Table 3:** Univariate linear regression between the total score of sexual health literacy and socio-demographic and other variables (N = 410).

Variables	B	P value	95% Confidence interval	
			Lower	Upper
Age	-0/20	**0/007** *	-0/36	-0/05
Education	4/3	**0/002** *	1/589	7/010
Education of fathers	4/03	**0/017** *	0/71	7/36
Education of mothers	2/39	0/243	-1/63	6/41
Job	-0/04	0/885	-0/63	0/54
Income	0/42	0/427	-1/32	2/18
Socioeconomic status	0/75	**0/001** *	0/29	1/21
Race	-6/74	**0/065**	-13/90	0/41
Disease history	-2/77	**0/039** *	-5/41	-0/13
Ability to identify reliable and non-reliable sources	-7/40	**<0/001** *	-9/20	-5/61
Having a sense of shame and embarrassment for obtaining sexual information	5/33	**<0/001** *	3/73	6/92
History of participating in sex education classes	3/05	**0/025** *	0/38	5/73
Obscenity of sexual issues in the family	4/85	**<0/001** *	3/23	6/48
Having the motivation to Obtain sexual information	-4/13	**<0/001** *	-5/73	-2/53
Satisfaction from genitalia shape and appearance	1/08	**<0/001** *	0/48	1/68
Satisfaction from sexual attractiveness	1/13	**<0/001** *	0/57	1/68
Believing that sexual problems are incurable	6/50	**<0/001** *	3/67	9/33
Sexual intimacy	0/40	**<0/001** *	0/31	0/49
Sexual self-efficacy	1/26	**<0/001** *	1/07	1/46
Marital intimacy	0/17	**<0/001** *	0/11	0/22
Social support	0/24	**<0/001** *	0/16	0/33
Sexual Knowledge and Attitude	0/48	**<0/001** *	0/40	0/55
Depression, Stress, Anxiety	-0/30	**<0/001** *	-0/39	-0/20

Bold*: Significant; B:Unstandardized Regression Coefficient

In the next step, multiple linear regression was used to determine the predictors of sexual health literacy, and variables with significant relationships from the univariate linear regression were entered into the model. At first, the variance inflation factor was used to check multicollinearity, which did not show any significant correlation between the variables in the final model (VIF <10). Based on the results of stepwise multiple linear regression, sexual self-efficacy (B = 0.649، P<0.001), sexual knowledge and attitude (B = 0.217، P<0.001), the ability to identify reliable and non-reliable sources (B = -3.116، P<0.001), feelings of shame and embarrassment for obtaining sexual information (B = 1.860، P = 0.011), social support (B = 0.127، P<0.001) and the obscenity of sexual issues in family (B = 1.764، P = 0.015) were the final predictors of sexual health literacy. These findings indicated that the sexual health literacy score increases by 0.649, 0.217, and 0.127 units for each unit increase in sexual self-efficacy, sexual knowledge and attitude, and social support, respectively, provided all other variables remain constant. Also, these results showed that those who don’t have the feeling of shame and embarrassment for obtaining sexual information and the obscenity of sexual issues in the family, their average of sexual health literacy score was 1.860 and 1.764 units higher, respectively. Moreover, those who don’t have the ability to identify reliable and non-reliable sources score, their average of sexual health literacy score was 3.116 units lower, provided all other variables remain constant. The results of multiple linear regression showed that the included variables explained 41.8% of the variance of the sexual health literacy score (R  =  0.653, R2  =  0.427, and ADJ.R2  =  0.418) (Table 4).

**Table 4:** The predictors of sexual health literacy in Iranian couples according to stepwise multiple linear regression (N = 410).

Variables	B	SE	P value	95% Confidence interval	
				Lower	Upper
(Constant)	19/51	5/47	**<0/001** *	8/75	30/27
Ability to identify reliable and non-reliable sources(no)	-3/11	0/81	**<0/001** *	-4/71	-1/51
Having a sense of shame and embarrassment for obtaining sexual information(no)	1/86	0/72	**0/011** *	0/43	3/28
Obscenity of sexual issues in the family(no)	1/76	0/72	**0/015** *	0/34	3/18
Sexual self-efficacy	0/64	0/11	**<0/001** *	0/42	0/87
Sexual Knowledge and Attitude	0/21	0/04	**<0/001** *	0/13	0/30
Social support	0/12	0/03	**<0/001** *	0/05	0/19

Bold*: Significant; B:Unstandardized Regression Coefficient; SE: Standard Error

## Discussion

This study aimed to determine sexual health literacy and its predictors among Iranian couples. The results of this study showed that the mean(SD) of sexual health literacy in couples was 68.76(12.960). Also, the results showed that sexual self-efficacy, sexual knowledge, and attitude, the ability to identify reliable and non-reliable sources, feelings of shame and embarrassment for obtaining sexual information, social support, and the obscenity of sexual issues in the family were the final predictors of sexual health literacy in couples.

Jamali et al. conducted a study in 2020 to determine sexual health literacy among women of reproductive age. The results of this study showed that the mean(SD) of sexual health literacy scores among reproductive-age women was 74.12)12.38) [12]. Dabiri et al. also conducted a survey in 2019 to determine sexual health literacy and fertility among couples about to get married. The results of this study showed that the mean(SD) of reproductive and sexual health literacy in men and women were 54(10) and 53(14), respectively [6]. The difference between the scores reported in the current study and the two mentioned studies can be caused by several factors, including the study populations and their characteristics, cultural and social context, the questionnaires used, and differences in other factors related to sexual health literacy.

The current study showed that sexual self-efficacy could increase sexual health literacy scores. The study’s findings by Wong et al. in 2021 could confirm the results of the current study. The results of their research showed that a sexual health literacy program could be effective in improving sexual self-efficacy [8]. According to Bandura (1977), achieving high self-efficacy beliefs is as important as having literacy skills. People who are competent and confident about their literacy skills will willingly perform the skills and solve problems [30]. Sexual self-efficacy focuses on concepts including control over one’s sexual life, an individual’s competence and ability as a sexual agent, ability to participate in safe sexual activity, suitability as a sexual partner, and ability to obtain sexual satisfaction [31]. In addition, the results of several studies have shown that people with higher sexual self-efficacy had a higher ability to seek treatment [32], solve sexual problems, and sexual self-care [33].

In addition, the present study’s results showed that feeling ashamed and embarrassed for obtaining sexual information was associated with a decrease in sexual health literacy score. Feeling shame and embarrassment is known as one of the obstacles to seeking treatment and solving sexual problems [34]. Based on the results of the study by Rakhshaee (2020), feelings of shame and privacy about sexual issues were one of the factors affecting sexual health literacy [35]. In a qualitative study, Jamali (2020) also investigated the factors affecting the sexual health literacy of women of reproductive age. The results of both studies aligned with the present study’s findings [36].

The results of the present study showed that social support had a positive effect on sexual health literacy. Thus, sexual health literacy was higher among participants with higher social support. Martin, in 2017, also proposed social support as one of the factors related to sexual health literacy, which was in line with the present study’s findings [4]. As social actors, people live in social environments that have varying degrees of support and resources. Social support can affect people’s health literacy by defining their access to resources and information, transfer of information, the effect of family and friends as sources for obtaining information, seeking treatment, and complying with medical care, and the effect of emotional support of the family on health literacy [37]. Therefore, the influence of spouses, family, friends, relatives, and other social networks should be considered in designing sexual health literacy interventions. Moreover, sexual health literacy interventions should consider individual differences in social support.

The results of the present study showed that the obscenity of sexual issues in the family had a negative effect on health literacy. In this way, an increase in the obscenity of sexual matters in the family was associated with a decrease in the score of sexual health literacy. In this regard, the study by Jamali (2020) presented the influence of the family as one of the effective factors on sexual health literacy among women of reproductive age. According to the results of their study, the traditionality of families, sexual taboos in the family, and considering discussing sexual issues in the family as unpleasant were among the negative factors that affected sexual health literacy [36]. Also, the role of the family was mentioned as one of the factors related to reproductive health literacy in the study by Dabiri on couples who were about to get married [38].

Another finding of the present study was that sexual knowledge and attitude were predictors of sexual health literacy. Jamali stated that sexual attitude was related to sexual health literacy [36]. Simpson (2015) believed that beliefs or attitudes play an important role in sexual health literacy, so a person’s attitude can provide a framework for the effectiveness of various sexual health literacy interventions [39]. The results of both studies confirmed the findings of the present study.

The present study was part of a comprehensive multi-stage study. To the best of our knowledge, no study has been conducted to determine the predictors of sexual health literacy among couples. Therefore, one of the strengths and novelty of the present study was the comprehensive and extensive study of sexual health literacy and its predictors among couples. The standard questionnaire, multi-stage random sampling method, and multiple regression model were among the positive aspects of the current research, which was conducted to increase internal and external validity. A limitation of the present study was the inability to perform the interviews with the participants in one session due to a large number of questionnaires. Therefore, to increase the credibility of the response and the participation of the couples, the questionnaires were completed in several stages. Another limitation was that the sampling method from comprehensive health service centers and private clinics could not be easily generalized to society.

## Conclusions

Based on the findings of the present study, sexual self-efficacy, sexual knowledge and attitude, ability to identify reliable and non-reliable sources, feelings of shame and embarrassment for obtaining sexual information, social support, and the obscenity of sexual issues in the family were the final predictors of sexual health literacy.

It is suggested that researchers and health managers consider these factors when designing interventions to promote sexual health literacy. Therefore, they should investigate effective strategies to improve sexual self-efficiency and promote sexual knowledge and attitudes, and also provide couples with education on identifying reliable and non-reliable sources and actionable solutions to reduce the feeling of shame and embarrassment for obtaining sexual information. Also, the influence of spouses, family, friends, relatives, and other social networks should be considered in designing sexual health literacy interventions. Moreover, sexual health literacy interventions should consider individual differences in social support.
